# Supplementary material for: Helical ultrastructure of the metalloprotease meprin α in complex with a small molecule inhibitor
Source: Nat Commun. 2022 Oct 19;13:6178. doi: 10.1038/s41467-022-33893-7 (PMC9581967; doi:10.1038/s41467-022-33893-7)
Supplement: Supplementary file 1 — Supplementary Info File #1 [file 41467_2022_33893_MOESM1_ESM.pdf]

SUPPLEMENTARY INFORMATION

Helical ultrastructure of the metalloprotease meprin α in complex with a small molecule inhibitor

Charles Bayly-Jones*[1,2], Christopher Lupton*[1,2], Claudia Fritz*[3], Hariprasad Venugopal[4], Daniel Ramsbeck[3], Michael Wermann[3], Christian Jäger[5], Alex de Marco[1,2], Stephan Schilling[3,6], Dagmar Schlenzig#[3], James C. Whisstock#[1,2,7,8]

[1]Biomedicine Discovery Institute, Department of Biochemistry and Molecular Biology, Monash University, Melbourne, Australia.

[2]ARC Centre of Excellence in Advanced Molecular Imaging, Monash University, Melbourne, Australia.

[3]Fraunhofer Institute for Cell Therapy and Immunology, Department for Drug Design and Target Validation (IZI-MWT), Halle, Germany.

[4]Ramaciotti Centre for Cryo-Electron Microscopy, Monash University, Clayton 3800, Victoria, Australia

[5]Vivoryon Therapeutics N. V., Halle, Germany

[6]Hochschule Anhalt, University of Applied Sciences, Köthen, Germany

[7]EMBL Australia, Monash University, Melbourne, VIC, 3800, Australia.

[8]ACRF Department of Cancer Biology and Therapeutics, John Curtin School of Medical Research, Australian National University, Canberra, ACT, 2601, Australia

*These authors contributed equally

#Correspondence to dagmar.schlenzig@izi.fraunhofer.de or james.whisstock@monash.edu

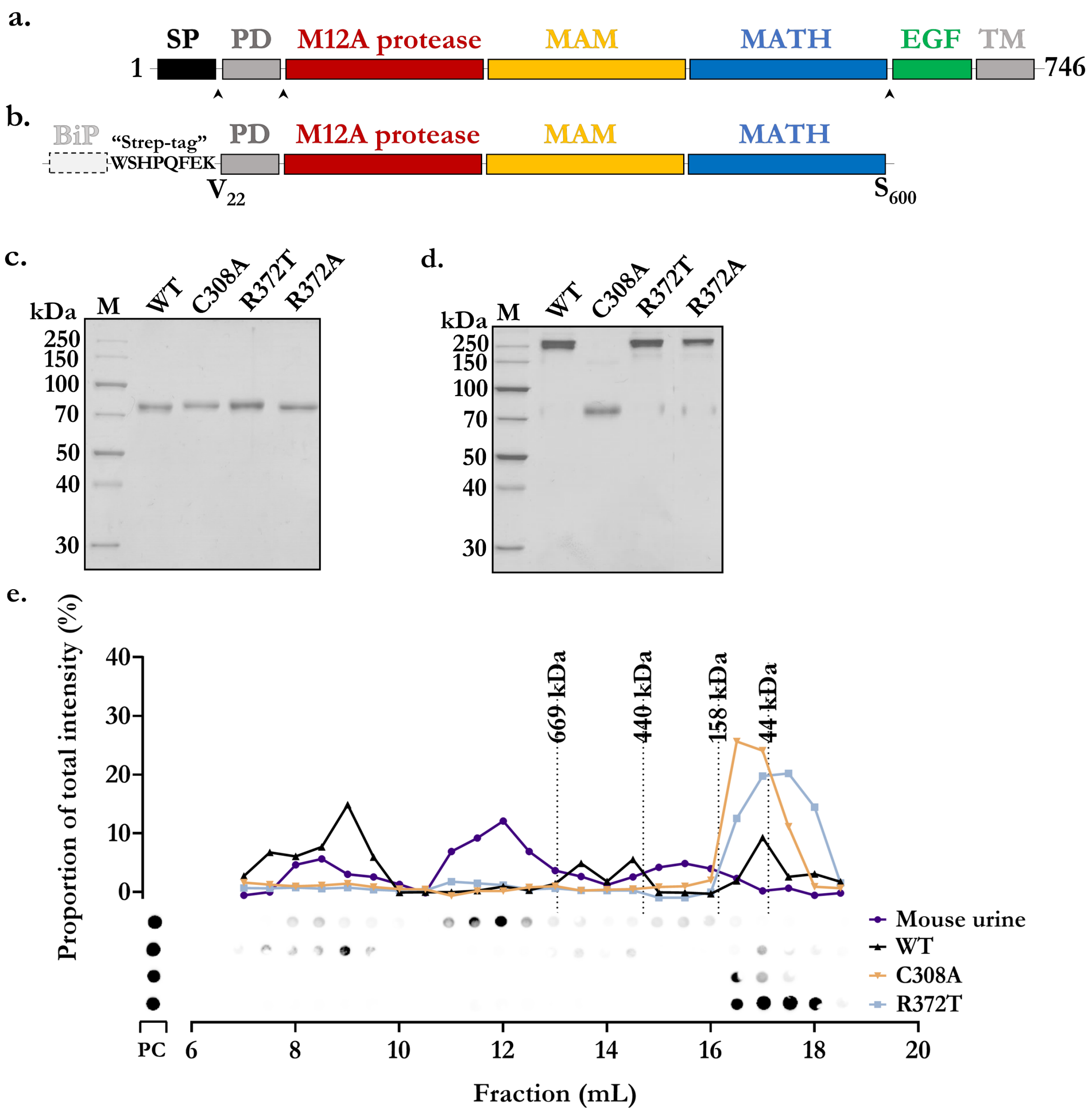

Supp. Figure 1. Meprin α construct expression, purification, and characterisation a. Domain schematic of native meprin α consisting of a signal peptide (SP), pro-domain (PD), proteolytic (M12A protease) domain, meprin, A-5 protein, and receptor protein-tyrosine phosphatase µ (MAM) domain, TNF receptor-associated factor (TRAF or MATH) domain, epidermal growth factor-like (EGF) domain and a transmembrane region (TM). Arrows indicate sites of proteolysis during normal meprin α maturation. **b.** Recombinant construct employed herein. Dashed boxes represent regions that are not genetically encoded in the recombinant construct. **c.** Reducing 10% SDS-PAGE analysis of purified recombinant meprin α and variants. **d.** As in (b) with non-reducing conditions. Protein purification was repeated at least n=5 times with similar results for (c) and (d). **e.** Comparison of retention volume (Superose® 6 10/300 GL; Cytiva) between recombinant meprin α (wild type, C308A and R372T) to native source meprin α isolated from mouse urine. Large oligomeric species were observed from the mouse urine after cisplatin treatment. These were, however, smaller than recombinant oligomeric meprin α. PC: injected material (i.e. pre-column) applied as a control. Trace shows immunoblot intensity. Meprin was detected by polyclonal antibody (R&D systems; human meprin α [#AF3220] and murine meprin α [#AF4007]). Theoretical molecular masses for globular well-behaved proteins are shown according to column calibration standards. Source data are provided as a Source Data file.

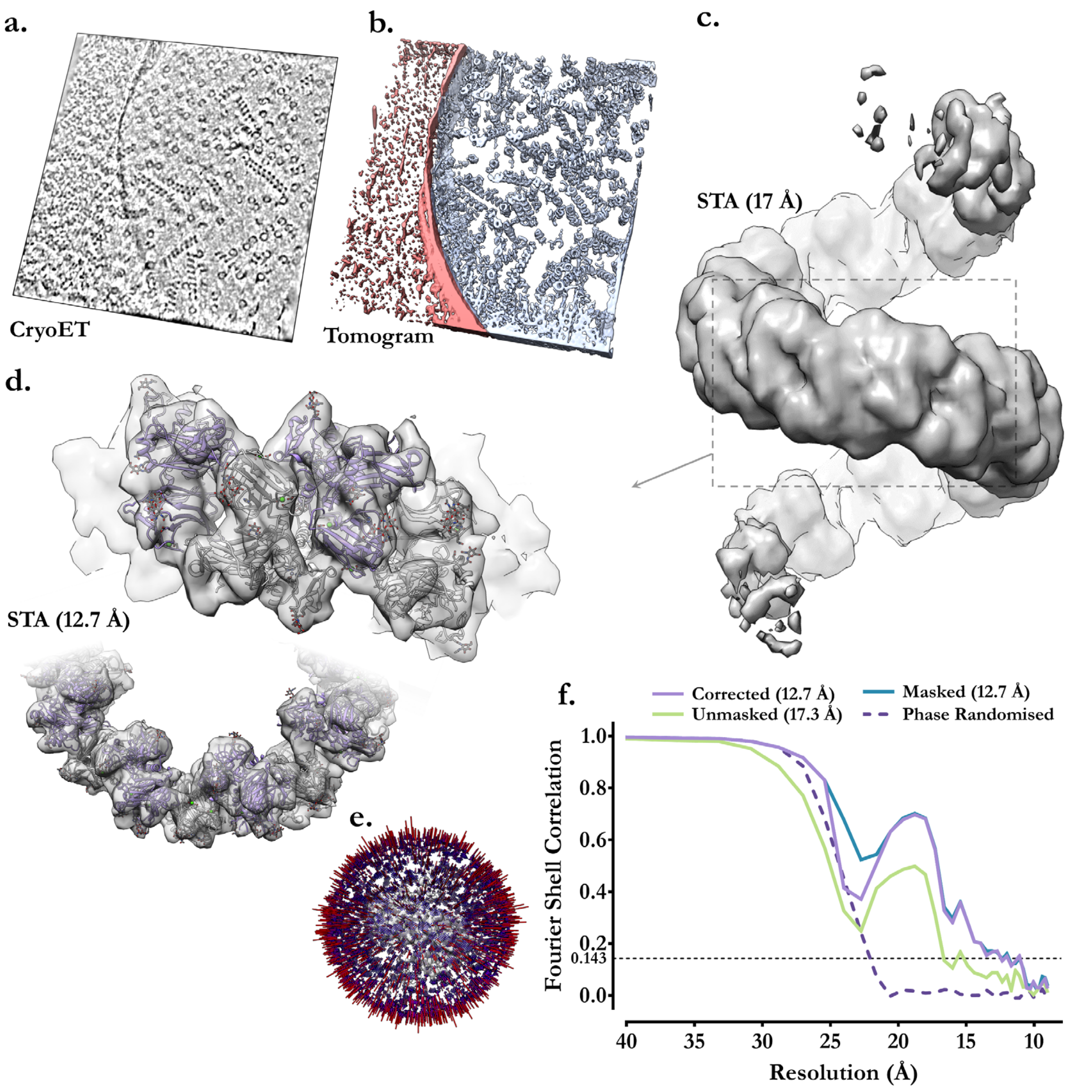

Supp. Figure 2. CryoET and sub-tomogram averaging of pro-meprin α filaments. a. A single z-slice of one cryotomogram and **b,** the corresponding surface representation low-pass filtered. **c.** A refined sub-tomogram average (STA) of meprin α filament including a full turn. Flexibility of the helix causes blurring at the edges. **d.** Two views of the sharpened STA corresponding to the final refined volume at 12.7 Å nominal resolution. Model to volume agreement is shown. **e.** Angular distribution of the final STA. **f.** Gold-standard FSC of the final STA.

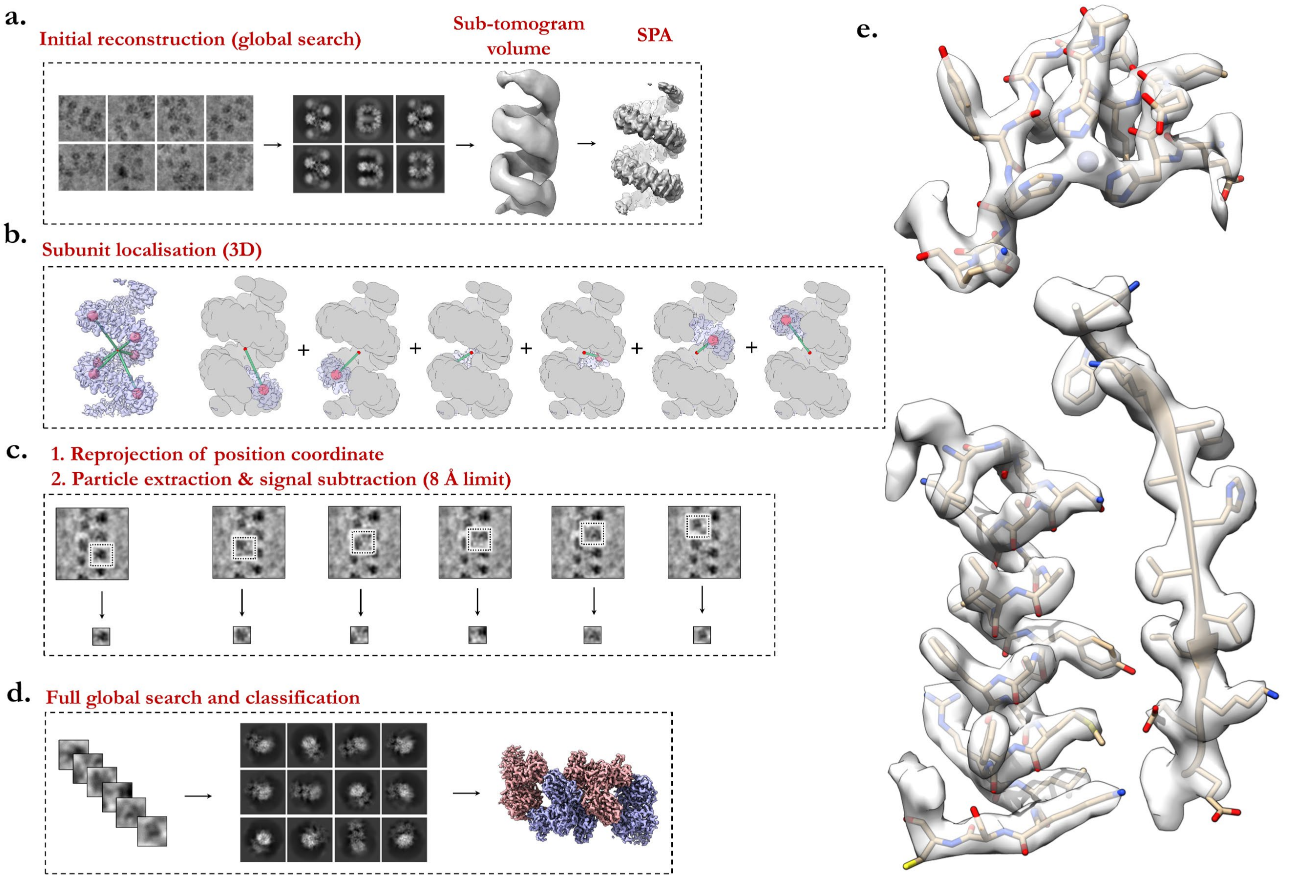

Supp. Figure 3. Summary diagram of cryoEM image analysis strategy and reconstruction method. a. Helical segments are broken into 20-30 nm fragments and classified independently. An initial volume was created by sub-tomogram averaging. Refinement of a full helix was performed by single-particle analysis. **b.** Coordinates of meprin α tetramers were defined based on the final reconstruction of the full helix and used to reproject 2D coordinates onto the original extracted particles. **c.** Sub-regions of meprin α tetramers were re-extracted from the original particles within a smaller area and subsequently treated as independent single particles. **d.** Assumption of single particle behaviour enabled reconstruction of a tetramer localised reconstruction by performing global searches with C1 "symmetry". **e.** Example regions of map and model agreement of the 2.4 Å meprin α reconstruction. Further details are available from the PDB and EMDB depositions (see Data availability).

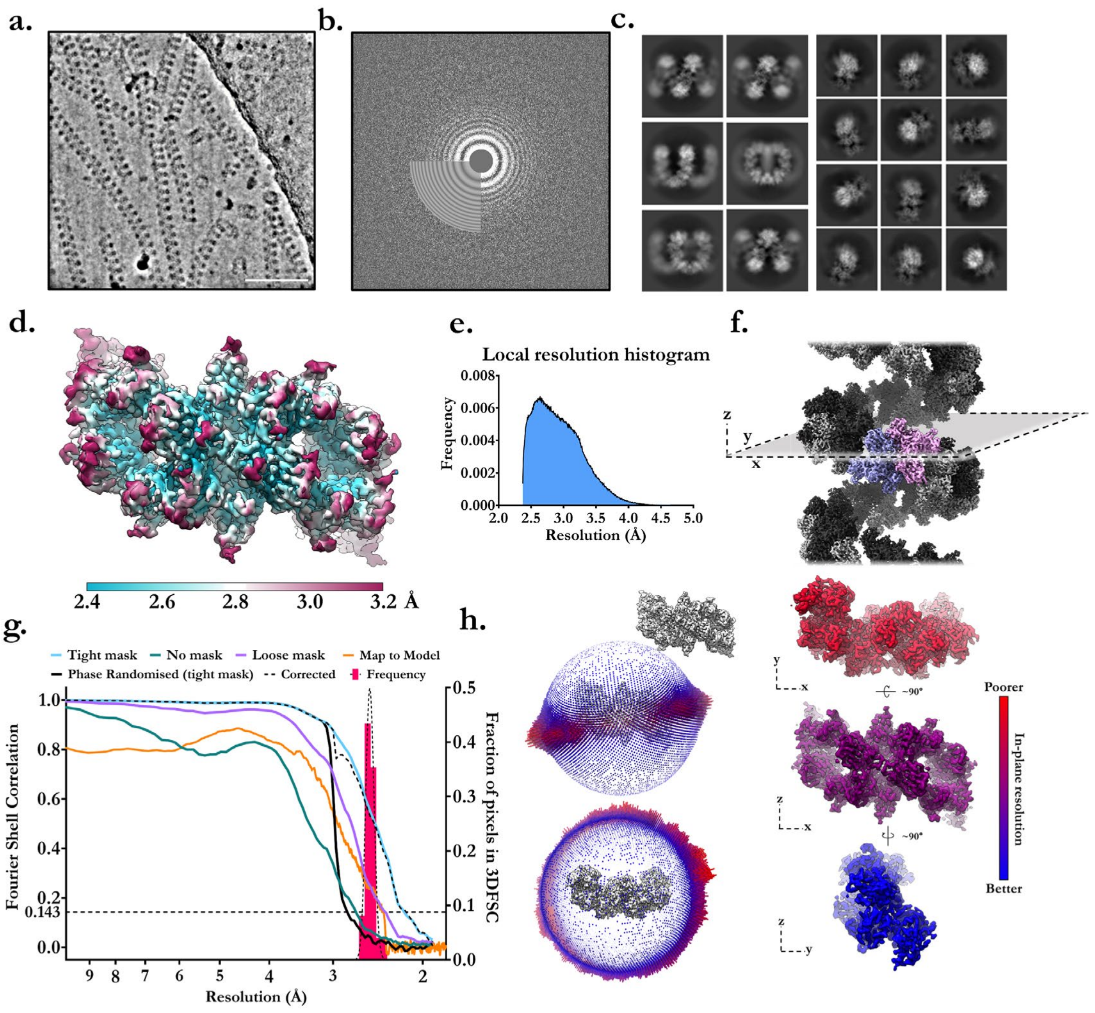

Supp. Figure 4. Summary figure of cryoEM key statistics and analysis outcomes. a. Representative micrograph of meprin α helices (scale bar: 100 nm) and, **b,** corresponding Fourier transform with high quality Thon rings. Similar experiments were conducted on six occasions with identical results. **c.** Class averages in 2D of meprin α helices and of meprin α tetramer subparticles after localised extraction. **d.** The final meprin α tetramer reconstruction coloured by local resolution. **e.** Corresponding per-voxel resolution frequency distribution. **f.** Composite map of the meprin α helix and corresponding directional resolution analysis. Maximum anisotropy is observed from the z-direction due to the tendency of helices to lie flat within the ice. **g.** Fourier shell correlation and directional resolution histogram plots. **h.** Angular distribution and orientation assignment of observed particles after refinement.

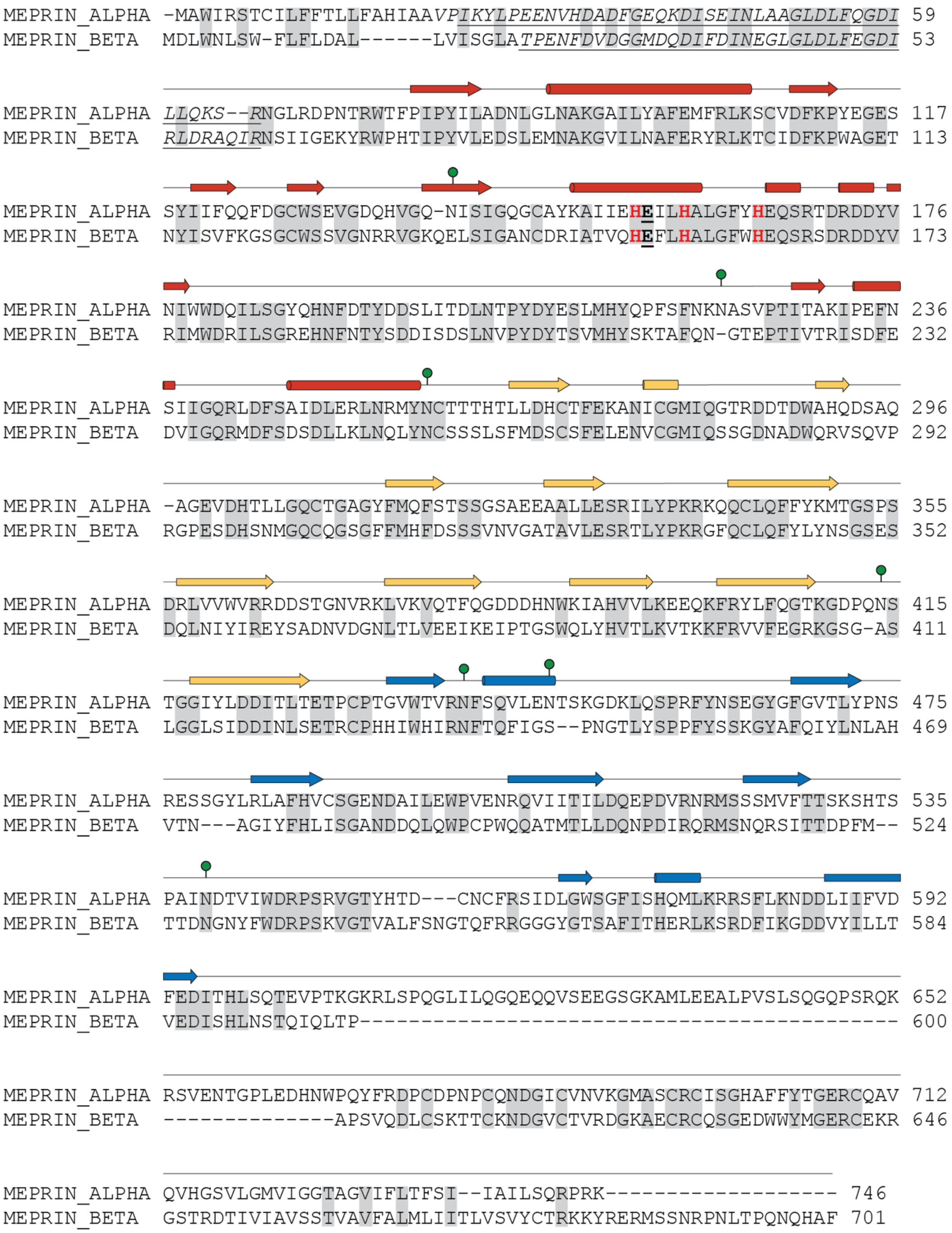

Supp. Figure 5. Sequence alignment of meprin α and meprin β. Secondary structure elements are shown above based on the structure reported herein, arrows represent β-strands and tubes represent α-helices. Green circles represent N-linked glycosylations. The active site triad of histidine residues are marked in bold font and red. The catalytic glutamic acid is black bold font emphasised.

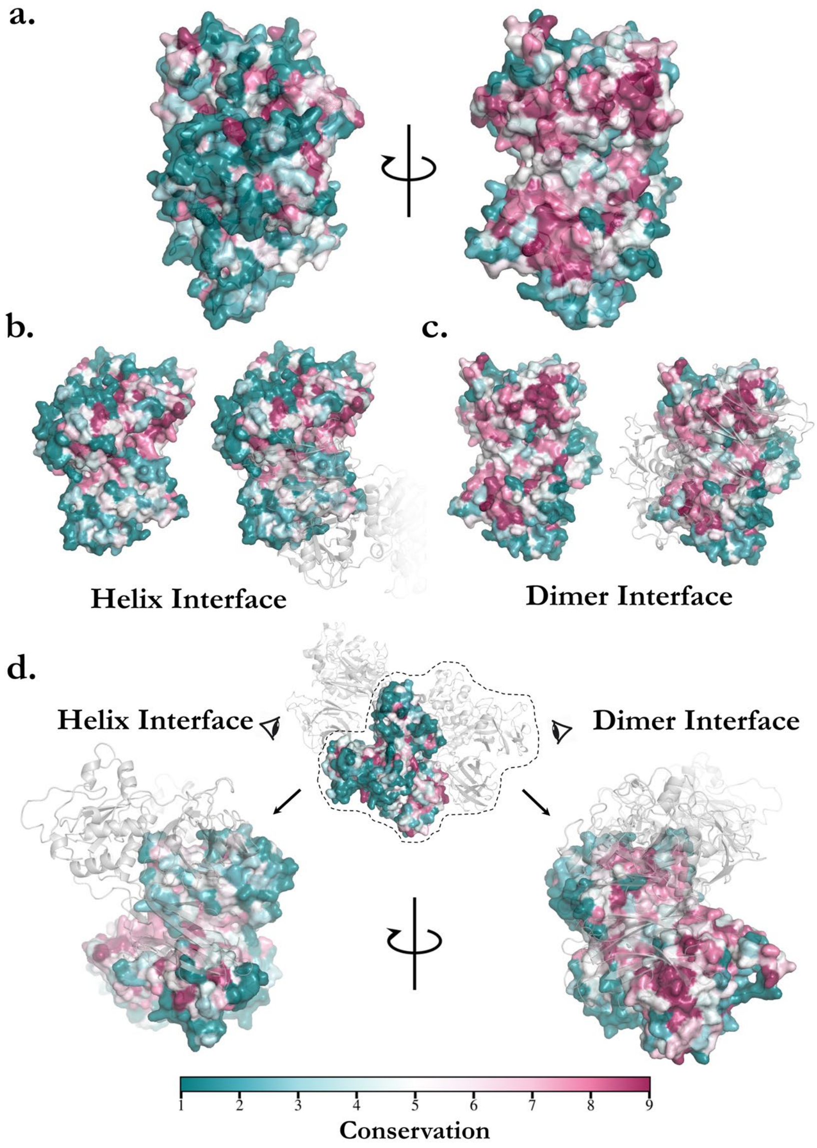

Supp. Figure 6. ConSurf analysis of meprin α dimer and oligomer interfaces. a. Monomer of meprin α with conserved surface rendering showing highly conserved dimeric interface (right). **b**. Focus of helical interface as conserved surface rendering (with adjacent monomer in transparent cartoon), showing conservation to some degree. **c.** Focus of dimer interface (with cartoon model of adjacent monomer in transparent), showing strong conservation corresponding to the binding site of the adjacent monomer. **d.** Single monomer of meprin α shown in context of the helical oligomer.

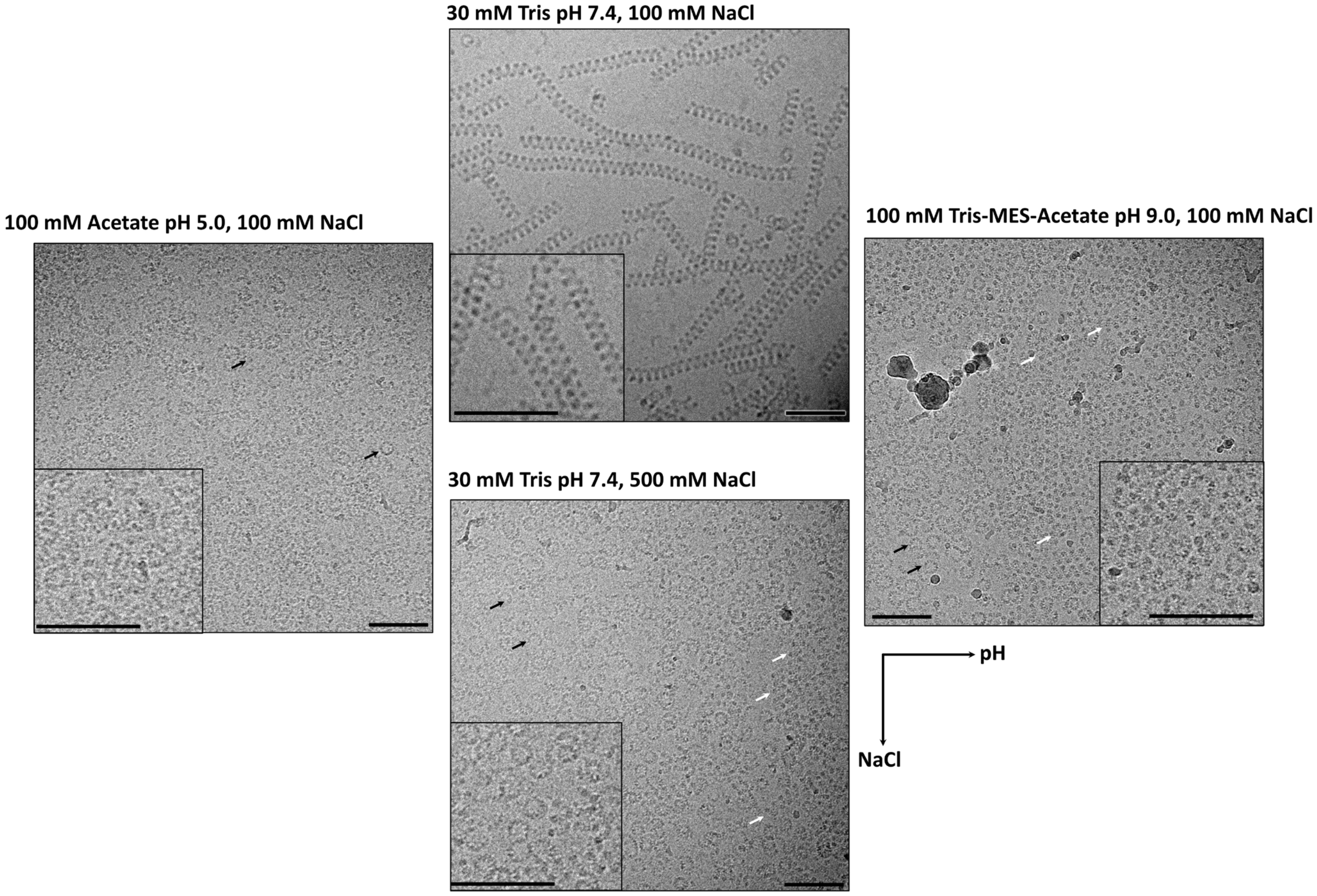

Supp. Figure 7. Comparison between meprin α appearance by cryoEM under different salt and pH conditions. Acidic conditions appeared to cause oligomer disassembly as observed by a greater proportion of dimeric and small oligomeric species. High concentration of NaCl (500 mM) has a similar impact. Conversely, the population of small oligomeric species at pH 9.0 were the minority, with prevalent supercoiled meprin oligomers observed. Comparisons are made against standard conditions of 100 mM NaCl and pH 7.4 Tris buffer. Black arrows depict dimers or small oligomers, white arrows show the presence of supercoiled meprin oligomers. High salt conditions were assessed on two separate occasions, low and high pH were assessed across a range, over multiple grids, and ice thicknesses. Scale bar (black) 100 nm.

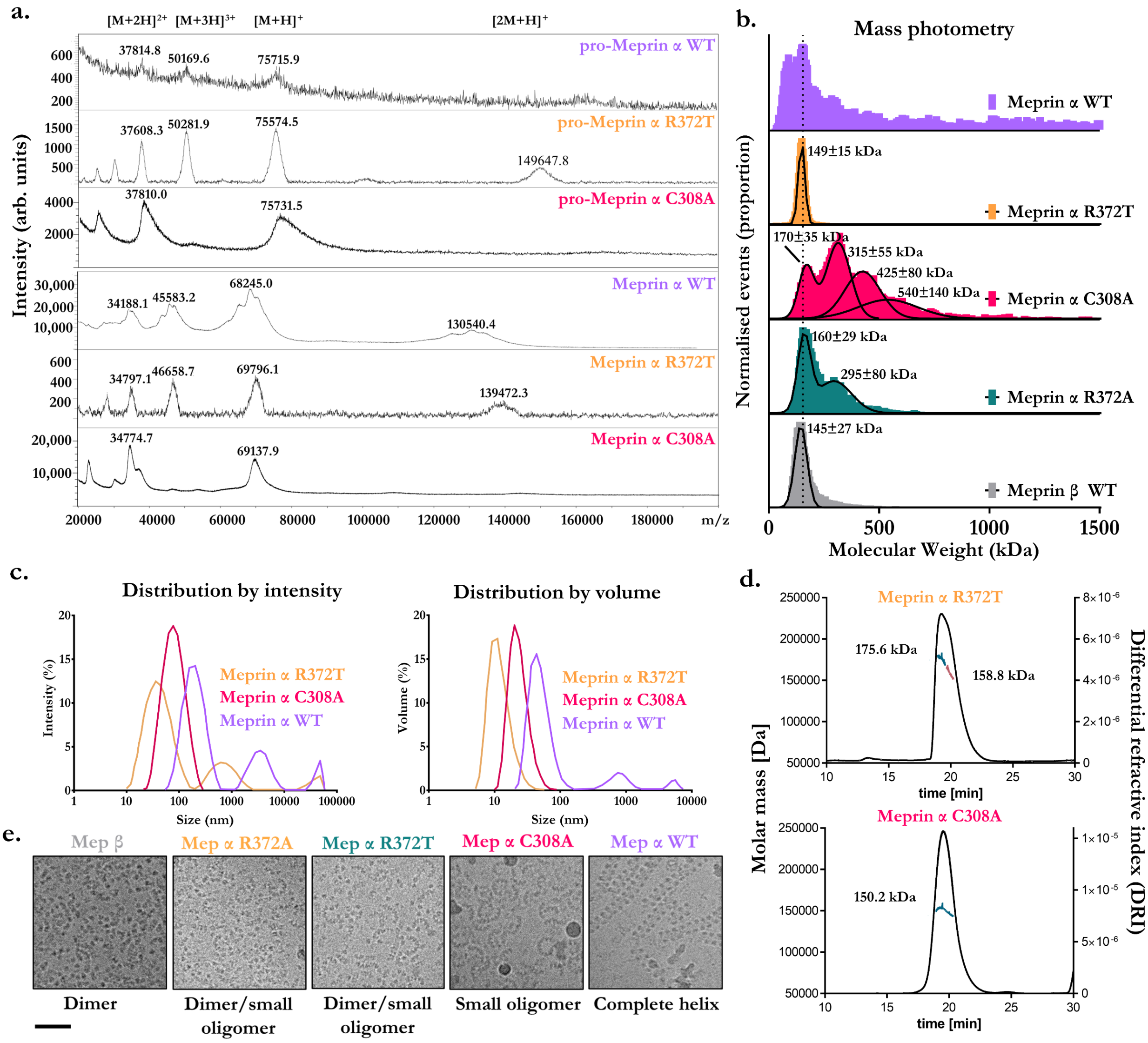

Supp. Figure 8. Biophysical characterisation of wild type meprin α and mutants C308A, R372T. a. MALDI-TOF analysis of wild type meprin α, C308A and R372T, as both zymogen and mature form. Peaks of about 75 kDa (zymogen) and 69 kDa (mature form) equal $[M+H]^+$ or $[M+2H]^+$, peaks of about 38 kDa (zymogen) and 35 kDa (mature form) equal $[M+2H]^{2+}$. **b.** Mass photometry single-molecule mass distribution of meprin α, β and variants. The mass distribution of wild type meprin α appears skewed toward smaller oligomeric species, however this is artificial since the largest filaments are excluded during event detection. Nevertheless, the highly polydisperse nature of the wild-type sample is apparent. **c.** Comparative analysis of hydrodynamic radius by MADLS of meprin α and mutants C308A and R372T with the Zetasizer Ultra. Overlay of intensity particle size distribution (left). Overlay of volume particle size distributions results are presented as the average value of three to five experiments (right). **d**. SEC-MALS analysis of pro-meprin α variants C308A and R372T. A homogenous peak for C308A was observed, while for R372T a heterogenous peak was detected, including two forms of dimeric pro-meprin α R372T. **e.** Cryo-TEM (200 kV) of meprin α and β, and mutant variants of meprin α. Repeated on three occasions, smaller species tend to be found in thinner ice while larger oligomers are found in thicker ice. Scale bar (black) 50 nm.

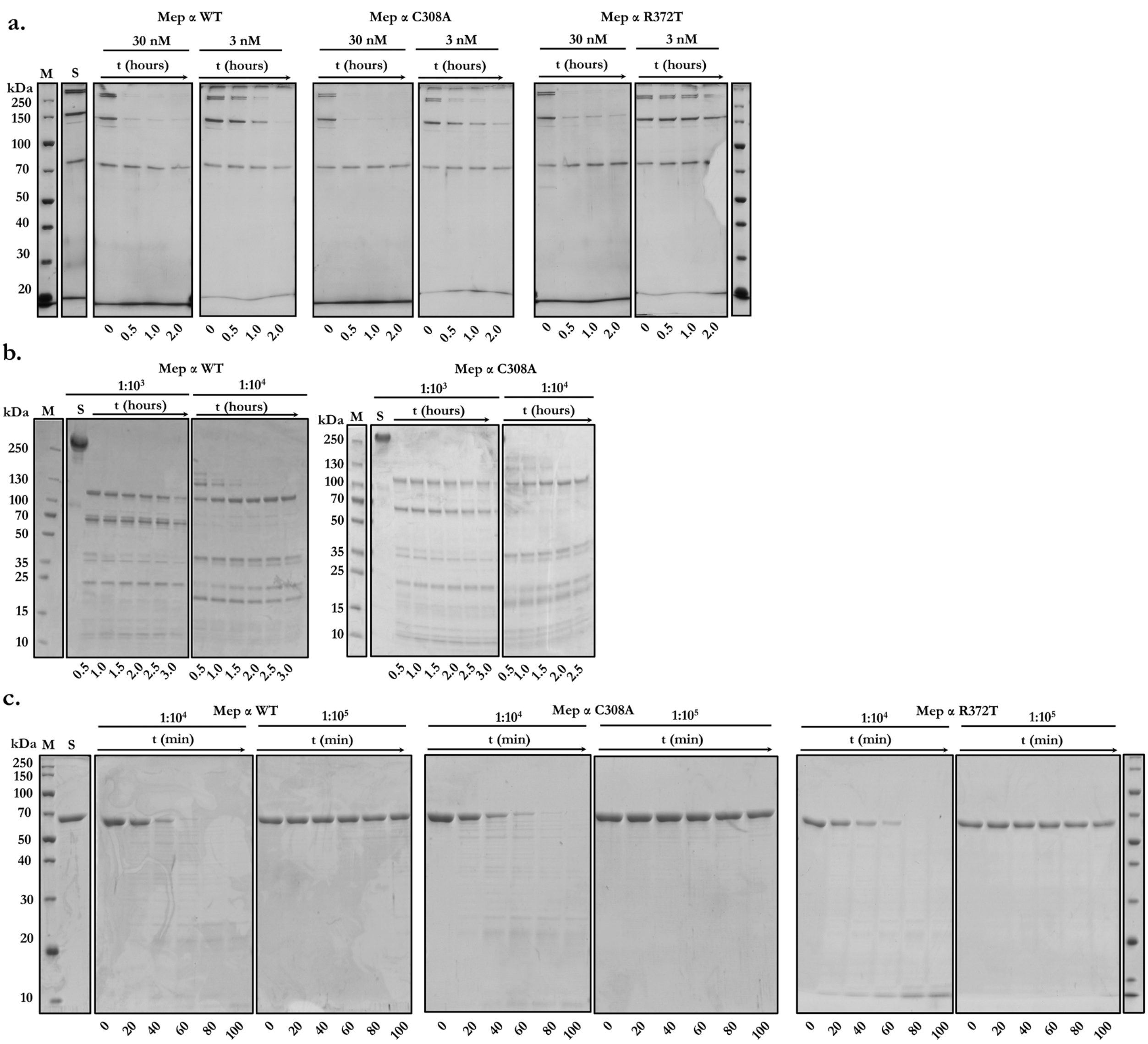

Supp. Figure 9. Meprin α substrate degradation and activity by variants and wild type. Oligomeric state does not appear to drastically affect substrate specificity or rate of degradation. **a.** Cleavage of rat tail procollagen by wild-type meprin α, C308A and R372T. Specific cleavage of procollagen by each meprin α variant results in similar cleavage patterns. Samples analysed using reducing 12% (w/v) SDS-PAGE, visualized by Coomassie-staining. **b.** Cleavage of human fibronectin by wild-type meprin α and variant C308A. Specific cleavage of fibronectin by both meprin α variants result in same cleavage pattern. **c.** Cleavage of human tropoelastin by wild type-meprin α, C308A and R372T. Complete degradation of tropoelastin by meprin α (regardless of variant) within 2 h (molar ratio of 1:10^4). Samples analysed using reducing SDS-PAGE (4-20% (w/v) gradient gel), visualized by Coomassie-staining. In each sample 10 µg of substrate were applied, as well as a control of substrate only (S; substrate incubated for 3 h at 37°C in the absence of meprin α). Source data are provided as a Source Data file. Substrate degradation assays were performed numerous times (n>4) with different batches of protein on separate occasions with similar representative outcomes.

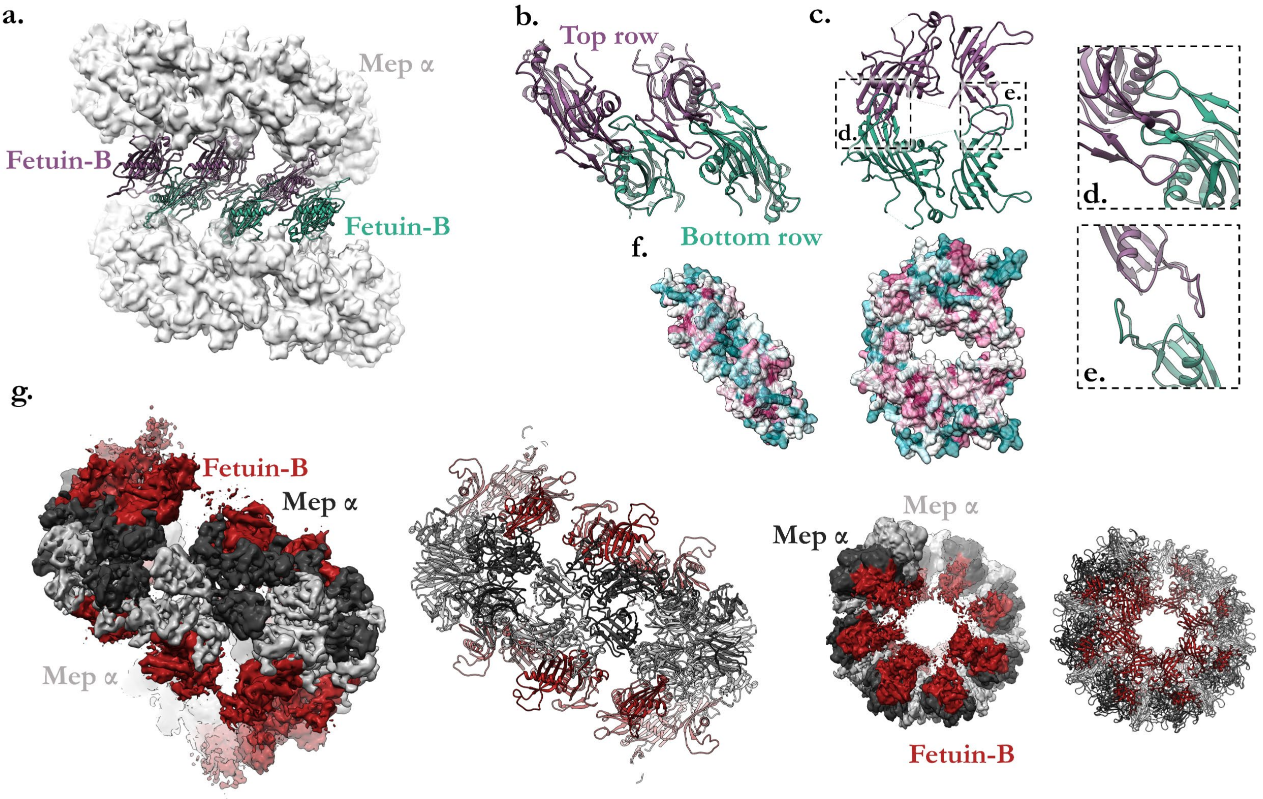

Supp. Figure 10. Molecular docking and 3.7 Å cryoEM reconstruction of fetuin-B inhibitor bound to meprin α. a. Rigid body fit of murine fetuin-B/meprin β crystal structure (PDB 7AUW) to helical structure of meprin α. Arrangement of fetuin-B reveal monomers may pack into a slanted intercalated state that is not significantly prohibited by steric clashes. **b.** View of a tetramer of fetuin-B based on meprin α docking reveals potential interactions to form a higher-order inhibitory filamentous complex are possible. **c.** Side view of single fetuin-B dimer forms a "horseshoe" where putative interactions between inter-subunit fetuin-B domains may occur shown in (**d**), and (**e**). **d, e.** Models are not refined, rigid body fitting results in some minor clashes. **f.** The predicted oligomeric interface corresponds to an evolutionarily conserved interface revealed by ConSurf analysis. **g.** Side and top views of the cryo-EM reconstruction of human fetuin-B (red) in complex with meprin α (grey, black) at 3.7 Å resolution. Fetuin-B is observed to pack intimately within the meprin α active groove and intercalate as a secondary helix.

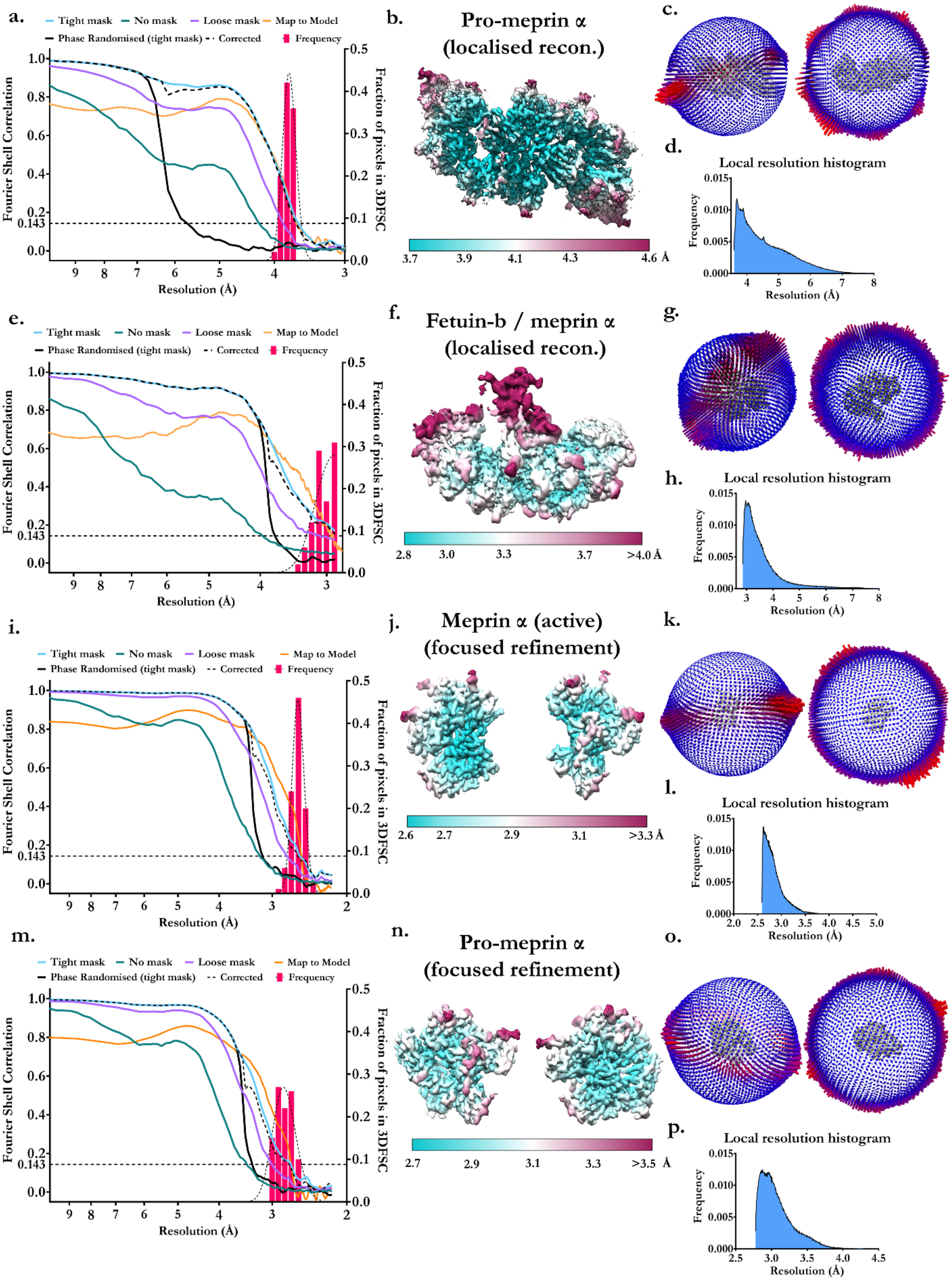

Supp. Figure 11. Summary figure of cryo-EM key statistics and analysis outcomes (zymogen, active and fetuin-B maps). a. Fourier shell correlations including tight, loose and no mask curves, phase randomised half-maps, mask corrected FSC, map-to-model FSC and histogram of directional 3DFSC voxels (frequency). **b.** Final reconstruction coloured by local resolution. **c.** Corresponding per-voxel resolution frequency distribution. **d.** Angular distribution and orientation assignment of observed particles after refinement. **e.—p.** As in a., b., c., d.

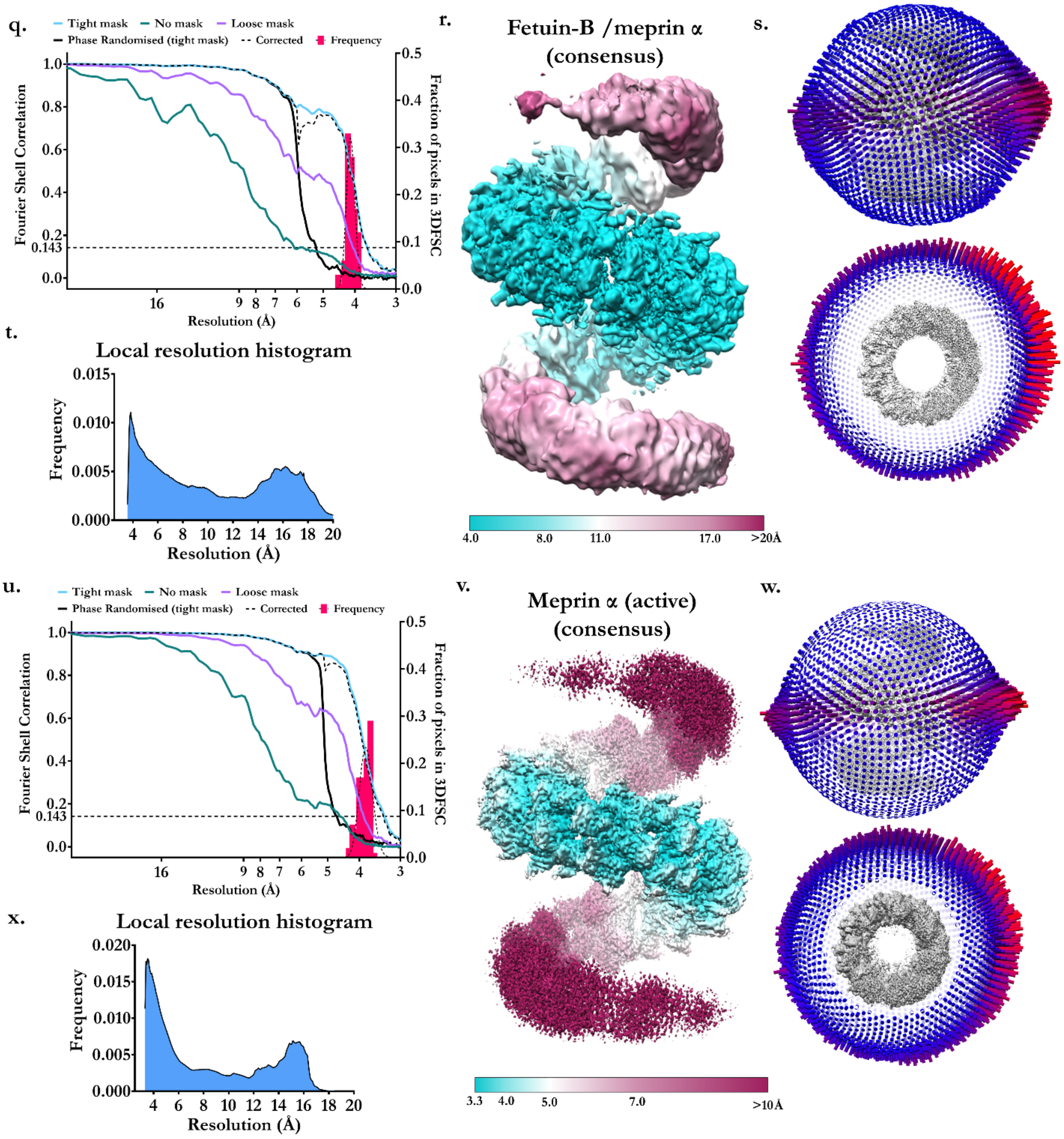

Supp. Figure 11 cont. Summary figure of cryo-EM key statistics and analysis outcomes (full helical active and fetuin-B maps). q. Fourier shell correlations including tight, loose and no mask curves, phase randomised half-maps, mask corrected FSC and histogram of directional 3DFSC voxels (frequency). **r.** Final reconstruction coloured by local resolution. **s.** Angular distribution and orientation assignment of observed particles after refinement. **t.** Corresponding per-voxel resolution frequency distribution. **u., v., w., x.,** as in q, r, s, t.

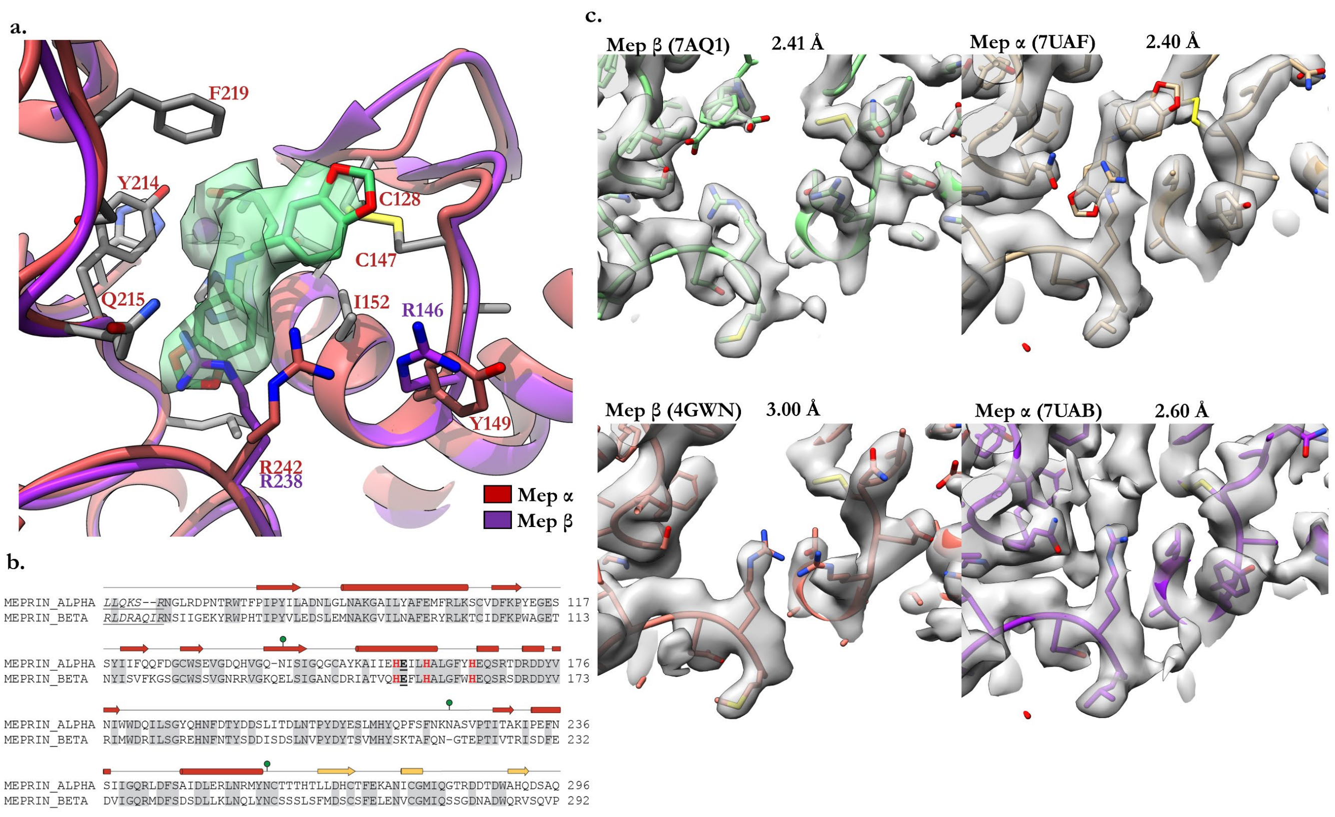

Supp. Figure 12. Structural comparison of meprin α and meprin β within the active site. a. Superposition of meprin α (red) and meprin β (purple) with select conserved side chains (grey) and divergent side chains (coloured) visible. **b.** Sequence alignment of meprin α and meprin β with secondary structure elements shown above (β-strands as arrows or α-helices as tubes). The catalytic glutamic acid is bold font and underlined. The active site tirade of histidines is shown in red bold font. Meprin α residue Y149 (meprin β R146) is non-conserved therefore presenting either as tyrosine or arginine in meprin α and meprin β respectively. This single residue appears to drive charge repulsion of R238 in meprin β (R242 in meprin α) causing a different rotamer position to be adopted relative to meprin α. This alternative rotamer conformation sterically occludes compound 10d (green; density shown as isosurface) suggesting how the drug remains selective despite major contacts of the drug and meprin α being conserved across both homologs. Conversely, in meprin α R242 is not repelled by Y149 and therefore adopts a conformation that does not interfere with drug binding. **c.** Experimental densities of the meprin α and β active site showing surface rendering and corresponding modelled site chains and rotamers.

Supp. Table 1 | Cryo-EM data collection, refinement, and validation statistics

	**Pro-Meprin α** (EMD-26419) (PDB 7UAB) **Dataset-180314**	**Pro-Meprin α** (EMD-26420) (PDB 7UAC) **Dataset-190220**	**Meprin α (helix)** (EMD-26421) **Dataset-190220**	**Meprin α** (EMD-26422) (PDB 7UAE) **Dataset-190220**	**Meprin α / Compound 10d** (EMD-26423) (PDB 7UAF) **Dataset-190218**	**Meprin α / Fetuin-B (helix)** (EMD-26424) **Dataset-220125**	**Meprin α / Fetuin-B** (EMD-26426) (PDB 7UAI) **Dataset-220125**	**Pro- Meprin α** (EMD-27689) **Dataset-180304**
**Data collection and processing**								(5 tomos)
Magnification	130,000×	130,000×	130,000×	130,000×	130,000×	81,000×	81,000×	64,000×
Voltage (kV)	300	300	300	300	300	300	300	300
Electron exposure ($e^-/Å^2$)	22.25	44.5	44.5	44.5	44.5	44.5	44.5	~100
Defocus range (μm)	−0.5 to −2.2	−0.5 to −1.5	−0.5 to −1.5	−0.5 to −1.5	−0.5 to −1.2	−0.5 to −2.0	−0.5 to −2.0	−2.8 ± 0.3
Pixel size (Å)(binned)	1.06	1.06	1.06	1.06	1.06	1.06 (1.4133)	1.06 (1.4133)	2.25 (4.5)
Symmetry imposed	C1	C1	C1	C1	C1	C1	C1	-44.1°, 21.2 Å
Initial particle images (no.)	580,400	905,660	147,823	905,660	925,458	132,975	519,760	1,625
Final particle images (no.)	116,080	338,429	111,312	233,978	235,162	103,952	115,951	1,573
Map resolution (Å) 0.143/0.5 FSC threshold	3.7 / 4.1	2.7 / 3.3	3.4 / 3.9	2.6 / 3.0	2.4 / 2.8	3.7 / 4.2	2.8 / 3.7	12.7 / 25.0
Map resolution range (Å) 0.5 FSC threshold	3.6 - 7.0	2.8 – 4.2	3.3 - 18.0	2.6 – 3.9	2.4 – 4.6	3.5 – 22.2	2.9 – 8.1	11 – 25
3DFSC sphericity 0.5 FSC threshold	0.98	0.96	0.87	0.97	0.98	0.86	0.98	0.81
Reconstruction type	Subparticle localised	Subparticle + 3D masked refinement	Consensus	Subparticle + 3D masked refinement	Subparticle localised	Consensus	Subparticle localised	Subtomogram average
**Refinement**								
Initial model (PDB code)	4GWN / AF-Q16819-v2	7UAB		7UAB	4GWN		AF-Q9UGM5-v2, 7UAB, 7AUW	
Model resolution (Å) 0.5 FSC threshold	3.8	3.0		2.8	2.7		3.4	
Map sharpening *B* factor* ($Å^2$)	−85	−103		−94	−57		−54	
Model composition								
Non-hydrogen atoms	16,877	4,540		4,513	17,864		21,938	
Protein residues	2,042	546		535	2,131		2,690	
B factors ($Å^2$)								
Protein	80.38	85.46		71.06	39.92		64.97	
Ligands	99.52	100.47		94.18	61.79		91/12	
R.M.S. deviations								
Bond lengths (Å)	0.012	0.012		0.011	0.012		0.013	
Bond angles (°)	1.722	1.781		1.742	1.801		1.777	
Validation								
MolProbity score	1.09	0.88		0.82	1.01		1.16	
Clashscore	1.07	0.57		0.11	1.19		1.32	
Poor rotamers (%)	0.44	0.00		0.00	0.16		0.90	
Ramachandran plot								
Favored (%)	95.96	96.84		96.06	96.93		95.82	
Allowed (%)	3.99	3.16		3.94	3.07		4.24	
Disallowed (%)	0.05	0.00		0.00	0.00		0.04	

*As determined by the Rosenthal and Henderson method[96]. For model building, amplitude corrected maps and deepEMhancer (which does not generate a *B*-factor) were used in combination. Where applicable, these maps have been deposited to the EMDB under the above codes.

Supp. Table 2 | DNA oligonucleotides used for site directed mutagenesis.

pMTB MepA_Strep (NcoI)	5'-ATACCATGGTGGTCCCACCCCCAGTTCGAGAAGGTACCGATTAAGTATCTTC-3'
pMTB MepA600 (NotI)	3'-GATATCACCCACCTCAGCTAAGCGGCCGCATATAT-5'
hMepA_C308A_f	ACACCTTGTTGGGACAAGCCACAGGTGCCGGCTACTTC
hMepA_C308A_r	GAAGTAGCCGGCACCTGTGGCTTGTCCCAACAAGGTGT
hMepA_ R372T_f	AGCACAGGCAATGTTACCAAGTTGGTGAAGGTG
hMepA_ R372T_r	CACCTTCACCAACTTGGTAACATTGCCTGTGCT
hMepA_ R372A_f	AGCACAGGCAATGTTGCCAAGTTGGTGAAGGTG
hMepA_ R372A_r	CACCTTCACCAACTTGGCAACATTGCCTGTGCT
