# Supplementary material for: Helical ultrastructure of the metalloprotease meprin α in complex with a small molecule inhibitor
Source: Nat Commun. 2022 Oct 19;13:6178. doi: 10.1038/s41467-022-33893-7 (PMC9581967; doi:10.1038/s41467-022-33893-7)
Supplement: Supplementary file 2 — Reporting Summary [file 41467_2022_33893_MOESM2_ESM.pdf]

nature portfolio

Corresponding author(s): James C. Whisstock, Dagmar Schlenzig

Last updated by author(s): Sep 15, 2022

Reporting Summary

Nature Portfolio wishes to improve the reproducibility of the work that we publish. This form provides structure for consistency and transparency in reporting. For further information on Nature Portfolio policies, see our Editorial Policies and the Editorial Policy Checklist.

Statistics

For all statistical analyses, confirm that the following items are present in the figure legend, table legend, main text, or Methods section.

n/a	Confirmed	
☐	☒	The exact sample size (*n*) for each experimental group/condition, given as a discrete number and unit of measurement
☐	☒	A statement on whether measurements were taken from distinct samples or whether the same sample was measured repeatedly
☐	☒	The statistical test(s) used AND whether they are one- or two-sided<br>*Only common tests should be described solely by name; describe more complex techniques in the Methods section.*
☒	☐	A description of all covariates tested
☒	☐	A description of any assumptions or corrections, such as tests of normality and adjustment for multiple comparisons
☐	☒	A full description of the statistical parameters including central tendency (e.g. means) or other basic estimates (e.g. regression coefficient) AND variation (e.g. standard deviation) or associated estimates of uncertainty (e.g. confidence intervals)
☐	☒	For null hypothesis testing, the test statistic (e.g. *F*, *t*, *r*) with confidence intervals, effect sizes, degrees of freedom and *P* value noted<br>*Give P values as exact values whenever suitable.*
☒	☐	For Bayesian analysis, information on the choice of priors and Markov chain Monte Carlo settings
☒	☐	For hierarchical and complex designs, identification of the appropriate level for tests and full reporting of outcomes
☒	☐	Estimates of effect sizes (e.g. Cohen's *d*, Pearson's *r*), indicating how they were calculated

Our web collection on statistics for biologists contains articles on many of the points above.

Software and code

Policy information about availability of computer code

Data collection	CryoEM data was collected with SerialEM (v3.8) or EPU (v2). NanoDSF collected using PR.ThermControl (v2.1.2). MALDI-TOF was collected using flexControl (Compass software suite)(v3.4). SEC-MALS was performed using ASTRA-6. Kinetics data were collected by ReaderControl (v5.70 R5) / MARS (v4.01 R2). Mass photometry: Refeyn AquireMP (v2.5)
Data analysis	CryoEM analysis: IMOD, MotionCor2 (v.1.3.0), NovaCTF, Dynamo, CTFFIND (v4.1.13), Warp (v1.0.7), Cube (v1), crYOLO (v.1.8.1), RELION (v2.1, 3.1, 4.0b), cryoSPARC (v3.3.1), pyEM (v0.5), localised_reconstruction.py, SWISS-MODEL (2019), UCSF Chimera (v1.13), UCSF ChimeraX (v1.3), Coot (v0.9.2), ISOLDE (v.1.1), PHENIX (v1.17), MolProbity (v4.4), AlphaFold (v2), deepEMhancer (v1). GraphPad Prism (v9.2.0). Mass Spec: flexAnalyse (v3.4). Kinetics: MARS (v4.01 R2), Excel (v2208). NanoDSF: PR.ThermControl (v2.1.2). Mass photometry: DiscoverMP (v2.5).

For manuscripts utilizing custom algorithms or software that are central to the research but not yet described in published literature, software must be made available to editors and reviewers. We strongly encourage code deposition in a community repository (e.g. GitHub). See the Nature Portfolio guidelines for submitting code & software for further information.

Data

Policy information about availability of data

All manuscripts must include a data availability statement. This statement should provide the following information, where applicable:

- Accession codes, unique identifiers, or web links for publicly available datasets
- A description of any restrictions on data availability
- For clinical datasets or third party data, please ensure that the statement adheres to our policy

Cryo-EM maps are deposited in the Electron Microscopy Data Bank (EMDB), while atomic coordinates are available from the RCSB Protein Data Bank under the following accession codes. Pro-meprin α (tetramer), EMD-26419 [https://www.ebi.ac.uk/emdb/EMD-26419] and PDB-7UAB [https://www.rcsb.org/structure/7UAB].

Pro-meprin α (single subunit), EMD-26420 [https://www.ebi.ac.uk/emdb/EMD-26420] and PDB-7UAC [https://www.rcsb.org/structure/7UAC]. Full meprin α helix in the active state (C1 reconstruction), EMD-26421 [https://www.ebi.ac.uk/emdb/EMD-26421]. Meprin α in the active state (single subunit), EMD-26422 [https://www.ebi.ac.uk/emdb/EMD-26422] and PDB-7UAE [https://www.rcsb.org/structure/7UAE]. Meprin α in complex with small molecule inhibitor (tetramer), EMD-26423 [https://www.ebi.ac.uk/emdb/EMD-26423] and PDB-7UAF [https://www.rcsb.org/structure/7UAF]. Meprin α in complex with the native fetuin-B inhibitor (tetramer), EMD-26426 [https://www.ebi.ac.uk/emdb/EMD-26426] and PDB-7UAI [https://www.rcsb.org/structure/7UAI]. Full meprin α helix in complex with the native fetuin-B inhibitor (C1 reconstruction) EMD-26424 [https://www.ebi.ac.uk/emdb/EMD-26424]. Sub-tomogram average of pro-meprin α (12-subunits), EMD-27689 [https://www.ebi.ac.uk/emdb/EMD-27689]. The following atomic coordinates were used for analysis, PDB-4GWN [https://www.rcsb.org/structure/4GWN], AF-Q16819-v2 [https://alphafold.ebi.ac.uk/entry/Q16819], and AF-Q9UGM5-v2 [https://alphafold.ebi.ac.uk/entry/Q9UGM5]. Source data for figure 3 b-g, supplementary figures 1 c-e, and 9, are provided with this paper. Mutagenesis data was obtained from the COSMIC database, meprin α [https://cancer.sanger.ac.uk/cosmic/gene/analysis?ln=MEP1A] and meprin β [https://cancer.sanger.ac.uk/cosmic/gene/analysis?ln=MEP1B]. Sequence numbering refers to the full length meprin α available from UniProt (uniprot: Q16819)[https://www.uniprot.org/uniprotkb/Q16819/entry]. Any additional information and data are made available by request from the corresponding author.

Field-specific reporting

Please select the one below that is the best fit for your research. If you are not sure, read the appropriate sections before making your selection.

☒ Life sciences ☐ Behavioural & social sciences ☐ Ecological, evolutionary & environmental sciences

For a reference copy of the document with all sections, see nature.com/documents/nr-reporting-summary-flat.pdf

Life sciences study design

All studies must disclose on these points even when the disclosure is negative.

Sample size	All experiments were conducted in vitro. Individual mutants, protein assays or measurements were conducted with a minimum replica of n=3. This was chosen ad hoc. No statistical methods were used to predetermine sample size. All in vitro assays were conducted at very minimum for a sample size of n=3, in some cases (detailed in the manuscript) larger sample sizes were selected. Sample sizes were chosen according to the degree of variability to improve precision. Accuracy was assessed (where possible) by conducting independent measurements with different techniques, enabling similar measurements with different approaches. Additional biological (protein batch-to-batch) and technical repeats were conducted in contexts where sources of error (such as pipetting, concentrations, reagent variability) resulted in a large standard deviations. A minimum experimental replicate of n=3 ensured batch-to-batch variation was accounted for in the uncertainty estimates. A minimum of three (no real upper limit) is largely a practical decision, as each replicate (n=1) can take as long as a month to conduct (protein expression, purification, kinetic assays, microscope time, etc).
Data exclusions	Outliers were excluded in kinetic data where bubbles or plate reader errors caused large spikes. Otherwise no data were excluded.
Replication	All attempts at reproducing results were successful (experimental attempts detailed in figure legends, minimum three). Experiments were conducted multiple times, sometimes where experimental variation was large, additional repeats were conducted.
Randomization	Experimental groups were not involved, thus randomisation is not applicable. Randomisation was not performed, no clincal or cohort studies were conducted. All experiments were performed in vitro.
Blinding	Group allocation was not performed. Thus blinding was not performed. Experimental outcomes are not subjective and have been analysed by numerous authors.

Reporting for specific materials, systems and methods

We require information from authors about some types of materials, experimental systems and methods used in many studies. Here, indicate whether each material, system or method listed is relevant to your study. If you are not sure if a list item applies to your research, read the appropriate section before selecting a response.

Materials & experimental systems

n/a	Involved in the study	
☐	☒	Antibodies
☐	☒	Eukaryotic cell lines
☒	☐	Palaeontology and archaeology
☐	☒	Animals and other organisms
☒	☐	Human research participants
☒	☐	Clinical data
☒	☐	Dual use research of concern

Methods

n/a	Involved in the study	
☒	☐	ChIP-seq
☒	☐	Flow cytometry
☒	☐	MRI-based neuroimaging

Antibodies

Antibodies used	Polyclonal goat anti-Meprin α (R&D System) catalog #AF3220<br>Monoclonal mouse anti-Meprin α (R&D System) IgG2B Clone # 364312

Validation	Polyclonal goat anti-Meprin α (R&D System) catalog #AF3220:<br><br>Only used for dot immunoblots (supp figure 1).<br><br>"Detects human Meprin alpha Subunit/MEP1A in direct ELISAs and Western blots. In direct ELISAs, approximately 10% cross-reactivity with recombinant mouse MEP1A and recombinant human MEP1B is observed."<br><br>Immunogen: Mouse myeloma cell line NS0-derived recombinant human Meprin alpha Subunit/MEP1A Val22-Gln601 Accession # AAA21338. Tested against recombinant meprin alpha. Antigen affinity purified.<br><br>Monoclonal mouse anti-Meprin α (R&D System) IgG2B Clone # 364312:<br>"Detects human Meprin alpha Subunit/MEP1A in direct ELISAs and Western blots. In direct ELISAs and Western blots, no cross-reactivity with recombinant human MEP1B or recombinant mouse MEP1A is observed."<br>Immunogen: Mouse myeloma cell line NS0-derived recombinant human Meprin alpha Subunit/MEP1A Val22-Gln601 Accession # AAA21338. Tested against recombinant meprin alpha. Protein A or G purified from hybridoma culture supernatant<br><br>Bond, J.S. and Beynon, R.J. (1995) Protein Sci. 4:1247.<br>Stocker, W. et al. (1995) Protein Sci. 4:823.<br>Bertenshaw, G.P., et al. (2001) J. Biol. Chem. 276:13248.<br>Ishmael, F.T. et al. (2005) J. Biol. Chem. 280:13895.

Eukaryotic cell lines

Policy information about cell lines

Cell line source(s)	Schneider's Drosophila S2 cells (Thermo Fischer Scientific) Catalog number: R69007
Authentication	The cells were not authenticated.
Mycoplasma contamination	The cells were not tested for mycoplasma infection.
Commonly misidentified lines<br>(See ICLAC register)	No commonly misidentified lines were used in this study.

Animals and other organisms

Policy information about studies involving animals; ARRIVE guidelines recommended for reporting animal research

Laboratory animals	Urine was collected for 24 hours from a metabolic cage of wild type Mus musculus, strain BL6, mixed sex, mixed age - urine was simply used as a native source of protein for comparisons against recombinant material. No behavioral or mouse specific experiments were actually conducted.
Wild animals	The study did not involve wild animals.
Field-collected samples	The study did not involve samples collected from the field.
Ethics oversight	The animal experiment was approved by the responsible animal ethics committee of the state of Saxony-Anhalt, Germany (Landesverwaltungsamt Sachsen-Anhalt, Department of Consumer Protection and Veterinary Affairs, Halle (Saale), Saxony-Anhalt, Germany) under the following approval number: 42502-2-1473 MLU.

Note that full information on the approval of the study protocol must also be provided in the manuscript.
