# Supplementary material for: Helical ultrastructure of the metalloprotease meprin α in complex with a small molecule inhibitor
Source: Nat Commun. 2022 Oct 19;13:6178. doi: 10.1038/s41467-022-33893-7 (PMC9581967; doi:10.1038/s41467-022-33893-7)
Supplement: Supplementary file 3 — Description of Additional Supplementary Files [file 41467_2022_33893_MOESM3_ESM.pdf]

Description of Additional Supplementary Files:

Extended Data 1. Multiple sequence alignment in FASTA format of meprin α orthologs.

Extended Data 2. Raw COSMIC mutagenesis data and frequency analysis aligned to meprin α and β primary amino acid sequence.

Supplementary Movie 1. Overview movie illustrating cryotomography of meprin α, as well as high-resolution reconstructions of the zymogen form, active form, in complex with compound 10d, and in complex with fetuinB.

Supplementary Movie 2. The filamentous form of meprin α is highly flexible showing bending and twisting motions (shown as two variability modes from cryoSPARC 3D variability analysis).
